# Specific components of face perception in the human fusiform gyrus studied by tomographic estimates of magnetoencephalographic signals: a tool for the evaluation of non-verbal communication in psychosomatic paradigms)

**DOI:** 10.1186/1751-0759-1-23

**Published:** 2007-12-04

**Authors:** Yuka Okazaki, Andreas A Ioannides

**Affiliations:** 1 Department of Brain Science and Engineering, Graduate School of Life Science and Systems Engineering, Kyushu Institute of Technology, Kitakyushu-shi, Japan; 2 Laboratory for Human Brain Dynamics, Brain Science Institute (BSI), RIKEN, Wako-shi, Japan

## Abstract

**Aims:**

The aim of this study was to determine the specific spatiotemporal activation patterns of face perception in the fusiform gyrus (FG). The FG is a key area in the specialized brain system that makes possible the recognition of face with ease and speed in our daily life. Characterization of FG response provides a quantitative method for evaluating the fundamental functions that contribute to non-verbal communication in various psychosomatic paradigms.

**Methods:**

The MEG signal was recorded during passive visual stimulus presentation with three stimulus types – Faces, Hands and Shoes. The stimuli were presented separately to the central and peripheral visual fields. We performed statistical parametric mapping (SPM) analysis of tomographic estimates of activity to compare activity between a pre- and post-stimulus period in the same object (baseline test), and activity between objects (active test). The time course of regional activation curves was analyzed for each stimulus condition.

**Results:**

The SPM baseline test revealed a response to each stimulus type, which was very compact at the initial segment of main M_FG_170. For hands and shoes the area of significant change remains compact. For faces the area expanded widely within a few milliseconds and its boundaries engulfed the other object areas. The active test demonstrated that activity for faces was significantly larger than the activity for hands. The same face specific compact area as in the baseline test was identified, and then again expanded widely. For each stimulus type and presentation in each one of the visual fields locations, the analysis of the time course of FG activity identified three components in the FG: M_FG_100, M_FG_170, and M_FG_200 – all showed preference for faces.

**Conclusion:**

Early compact face-specific activity in the FG expands widely along the occipito-ventral brain within a few milliseconds. The significant difference between faces and the other object stimuli in M_FG_100 shows that processing of faces is already differentiated from processing of other objects within 100 ms. Standardization of the three face-specific MEG components could have diagnostic value for the integrity of the initial process of non-verbal communication in various psychosomatic paradigms.

## Findings

Faces constitute perhaps the most important stimuli in social interactions. Facial expression is one of the most informative non-verbal cues and it is analyzed soon after the initial process for non-verbal communication related to face perception is completed [1]. It is well-known that face perception is under the great influence of attention, emotional states and various neuro-endocrine conditions [2]. A quantitative analysis of the neural process of face perception may provide a tool to evaluate the fundamental functions of the initial step of non-verbal communication, which is impaired in certain psychosomatic patients. However, the results obtained so far are unclear about how face specificity develops in space and time.

Many fMRI studies have identified activations elicited by faces in compact ventral parts of the regions, in particular within the fusiform gyrus (FG) [3-5]. Other fMRI studies demonstrated that when information from a number of fMRI voxels is combined, a number of different visual stimuli, not just faces, elicit category specific responses [6-8]. Since the fMRI signal relies on hemodynamics, these results relate to slow processes, with a characteristic time-scale of seconds. Intracranial recordings provide information with high temporal resolution and spatial accuracy, but only at the locations dictated by clinical requirements. Such invasive measurements have identified a face-specific N200 that spread over ventral and temporal aspects of the brain [9]. MEG studies claimed face specific-responses in the FG for the M170 component [10,11]. A few studies have reported early face induced responses within 100 ms, some in early visual areas [10,12], while others were in the FG [13]. The last study did not use non-face control stimuli, so it could only demonstrate that face stimuli elicit early activity in FG, but could not determine whether this component was face-specific. What is therefore needed is accurate tomographic localization and precise timing in the measurement of face and non-face stimuli within the same experiment.

Seven, healthy right-handed Caucasian male subjects (mean age, 30.0 ± 5.0 years) gave informed, written consent, after the protocol was explained to them. The MEG protocol was approved by the Research Ethics Committee of RIKEN.

We used grayscale images of faces, hands and shoes. Shoe images were provided courtesy of the website [14]. Each stimulus was presented for 300 ms in one of five locations, either in the center or at 10.7° eccentricity from fixation across the diagonal. In each run, stimulus was presented five times in three of the five locations, alternating the choices to equally cover all five locations in the different runs. In central presentations, faces, hands, and shoes were presented at sizes of 5.5 × 4.1°, 4.5 × 3.6°, and 4.8 × 3.5° respectively. In the periphery, images were 8.2 × 6.1°, 6.7 × 5.3° and 7.1 × 5.2° in size respectively. The subject's task was to fixate on the central cross and to respond to the subtle change in its color with a quick button press.

Magnetic fields were measured with the MEG systems (Omega 151, CTF Systems Inc., Vancouver, B.C., Canada) in a magnetically shielded room (MSR). The Presentation software (Neurobehavioral Systems, Inc., Albany, CA) controlled a DLP projector with a 96 Hz refresh rate (HL8000Dsx+, NEC Viewtechnology Ltd., Tokyo, Japan) located outside the MSR. The exact onset time of each stimulus was determined by luminance detection with a photodiode on the screen. The EOG and ECG were simultaneously recorded and trials with eye movements or blinks exceeding 50 μV EOG signal change during the stimulus presentation period were discarded. Remaining artifacts were identified by strong ICA components correlated with either EOG or ECG and were removed. The signals from all channels were digitized at a sampling-rate of 625 Hz. The MEG signal was filtered with a bandwidth of 3 – 200 Hz and with notches at 50 Hz and its harmonics to eliminate power-line noise. The MEG sensors were determined relative to the individual subject MRI images for each run by the localization of fiduciary coils and our in-house co-registration procedure [15]. Magnetic field tomography (MFT) [16,17] was applied to each time slice of data (every 1.6 milliseconds apart) to extract independently tomographic estimates of neuronal activity.

For each subject, we performed voxel-by-voxel statistical parametric mapping (SPM) analysis by comparing the sample distribution for modulus of the MFT solutions, separately for each stimulus and VF. Two types of SPM analysis were performed, with Bonferroni-correction applied in each case to account for Type I errors due to multiple voxel comparisons. In the *active test *SPMs were produced by comparing the samples within a latency window (width = 19.2 ms) between two conditions. In the *baseline test *the comparison was between samples consisting of one sample from every three runs in the post-stimulus period and random samples from the pre-stimulus period (-250 ms to -50 ms). More details on SPM analysis can be found elsewhere [18].

To define regions of interest (ROIs) for the left and right FG in each subject, we used anatomical criteria – the collateral sulcus and the temporal occipital sulcus, and the SPM results of baseline test. The ROIs for central and contralateral peripheral presentations were defined independently of each other. We used circular statistics [19,20] to define the dominant direction of the MFT current density elicited by face stimuli in the 120 ms to 180 ms range inside the given ROI with a radius of 10 mm.

After ROI definition, a regional activation curve (RAC) was calculated for each stimulus condition at every time-slice by projection of the current density vector onto the dominant direction. Momentary amplitudes of the RAC were analyzed using ANOVA, following the same conceptual steps as for the SPM analysis, but with a 4.8 ms running window stepped every 1.6 ms. In the RAC active test, an ANOVA was performed with Stimulus type (Faces, Hands, Shoes), Hemisphere (left, right), and VF (upper, lower) as fixed factors, and Subject (seven subjects) as a random factor. Period (pre-stimulus, post-stimulus) was added as an additional fixed factor in the RAC baseline test.

**Figure 1:**
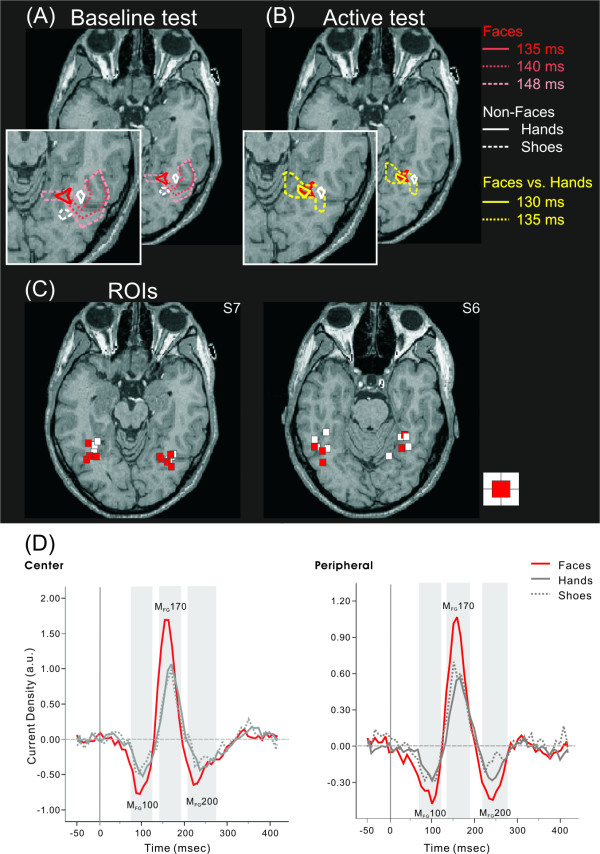
(A) Common activations from SPM baseline tests for central presentation. The contours delineate areas where the activity during the active period is higher (*p *< 0.05) than the baseline for at least 5 out of 7 subjects. Separate contours show the results for face (gradation red – different timing), hand (white solid) and shoe (white dash) stimuli. (B) Results displayed in the same format as in (A) but for the active test with contours delineating areas where the activity for face stimuli is higher (p < 0.005) than hands in all seven subjects (yellow). The baseline result for faces at 135 ms is copied from (A) and it can just be distinguished from the active test at 130 ms. (C) ROI centers (identified by the baseline test of) for all subjects, transformed via common Talairach coordinates to the space of the MRI display. Red and white squares mark the ROI centers of individual subjects for central and peripheral presentations. (D) The regional activation curves (RAC). RAC are averaged across subjects in the left and right FG for central and peripheral visual field presentation. The gray, shaded areas mark periods with activity higher than baseline (-250 ms – -50 ms). For both center and periphery, three components can be clearly seen: M_FG_100, M_FG_170, and M_FG_200.

Highly significant SPM foci for each object were intermittently identified from about 90 ms to 230 ms in FG. Face stimuli elicited activity within 100 ms. The contours in Figures 1A and 1B show the common significant FG activations across subjects around 130 – 150 ms for the baseline (*p *< 0.05) and active (*p *< 0.005) tests. For the baseline test the contours for Faces expanded within a few milliseconds and engulfed the other object area. Activity for non-face stimuli were consistently compact compared to face. The active test indicated significantly higher activity for Faces than Hands, over a wide area that included the area activated by Hands in the baseline test.

The subject specific ROIs as displayed in figure 1C were used to compute RAC. Figure 1D shows the grand averaged RAC across subjects for each stimulus type, separately for center and periphery. The RAC baseline test identified statistically significant FG activation across subjects in three periods. For central presentation these were at 71.0–121.0 ms (*F*(1, 6) = 47.6, *p *< 0.05), 135.0–186.0 ms (*F*(1, 6) = 22.4, *p *< 0.05), and 204.0–263.0 ms (*F*(1, 6) = 20.2, *p *< 0.05). For the peripheral presentation, significant activation periods were at 65.0–113.0 ms (*F*(1, 6) = 30.2, *p *< 0.05), 127.0–177.0 ms (*F*(1, 6) = 45.3, *p *< 0.05), and 207.0–263.0 ms (*F*(1, 6) = 35.3, *p *< 0.05).

We performed a post-hoc test (Tukey's method) for the most significant main effects of stimuli at the three identified peaks. No main effect of Hemisphere or Stimulus type × Hemisphere interaction was found. In the expected face selectivity at M_FG_170, the amplitude for the central presentation of face stimuli became significantly stronger than the other objects at 135.0 ms with a main effect of Stimulus type according to ANOVA (*F*(2, 12) = 8.76, *p *< 0.005), and showed an amplitude peak at 153.0 ms. Similarly, peripheral presentation showed a stronger response to faces at 126.0 ms (*F*(2, 12) = 7.11, *p *< 0.01), with an amplitude peak at 150.0 ms. Subsequent significant (M_FG_200) differences were found at 207.0 ms for the central presentations (*F*(2, 12) = 4.97, *p *< 0.05) and 246.0 ms for peripheral presentations (*F*(2, 12) = 13.81, *p *< 0.001). The analysis revealed that the response within 100 ms (M_FG_100) was also stronger for face stimuli compared to other objects, peaking at 73.0 ms (range, 63.0–81.0 ms), and 65.0 ms (range, 60.0–81.0 ms), for central (*F*(2, 12) = 13.15, *p *< 0.001) and peripheral presentations (*F*(2, 12) = 11.14, *p *< 0.05), respectively.

Our results resolve the apparent contradictions in previous fMRI and intracranial studies regarding object-selective responses in the ventral visual stream and add a critical temporal dimension to the analysis. In short, we found a compact response during the initial segment of the main component (M_FG_170). Compared to the baseline, the area of higher activity for hands and shoes remained compact, but the area for faces expanded widely and engulfed (just within its borders) the areas for hands and shoes. Direct comparison between faces and hands showed increased activity for faces, with a compact area identical to the early compact area of the face baseline test. This area expanded widely in the next few milliseconds. Thus our results show the compact face-specificity in the FG reported in some fMRI studies [3-5] during the early segment of each object-specific response. A few milliseconds later the compact face-specific activation in the FG expands, widely reproducing the pattern seen along the ventro-temporal cortex in other fMRI studies [6-8] and in studies with intracranial recording [9].

The time course for FG showed three components. The strongest peaked around 170 (M_FG_170) in agreement with many other studies [10,21,22]. An earlier component peaked within 100 ms (M_FG_100), and a later one after 200 ms (M_FG_200), again in agreement with previous studies [12,13]. Analysis of these time courses demonstrated a clear bias toward face stimuli for all three components. The face-selectivity of the early FG activity within 100 ms, demonstrated for the first time in our study, is particularly relevant to models attempting to explain how facial identity is processed with speed and accuracy.

In the present study, we found the three specific components of face perception in the FG area. Preliminary results from further analysis of our data suggest that the later components are modulated by attention. Detail analyses of M_FG_100, M_FG_170 and M_FG_200 in psychosomatic patients may reveal initial processes of face perception in non-verbal communication that are impaired in certain psychosomatic patients, especially patients with alexithymia [23,24].

## List of abbreviations

ECG: Electrocardiogram;

EOG: Electrooculogram;

fMRI: Functional magnetic resonance imaging;

ICA: Independent component analysis;

MEG: Magnetoencephalography.

## Authors' contributions

YO carried out the MEG experiment and performed analysis. AAI conceived of the study and participated in its design and coordination. The manuscript was drafted by both authors.
